# Editorial: Tailoring immunotherapy in gastrointestinal cancer: the role of circulating factors

**DOI:** 10.3389/fonc.2023.1260183

**Published:** 2023-08-08

**Authors:** Stefano Ugel, Chiara Bazzichetto, Fabiana Conciatori

**Affiliations:** ^1^ Immunology Section, Department of Medicine, School of Medicine and Surgery, University of Verona, Verona, Italy; ^2^ Preclinical Models and New Therapeutic Agents Unit, IRCCS Regina Elena National Cancer Institute, Rome, Italy

**Keywords:** gastrointestinal cancer, cancer immunotherapy, biomarkers, inflammation, immune system

Gastrointestinal (GI) cancer include malignant tumors affecting digestive organs such as esophagus, stomach, liver, pancreas, small intestine, colon and rectum. Its worldwide incidence and mortality are increasing in the last years, and the overall 5-years survival rate of patients remains less than 15%. In particular, with 1.9 million new cases and almost 0.9 million deaths in 2020, colorectal cancer (CRC) accounts for approximately 10% of all cancers and cancer-related deaths (1). Thus, prevention and treatment of GI cancer represent a major public health challenge. Moreover, the heterogeneous disease setting, challenges in early diagnosis, rapid-acting cancer development, and induction of resistance to therapy ascertain the need to identify diagnostic, prognostic and predictive biomarkers with innovative approaches for GI cancer (**Figure 1**).

**Figure 1:**
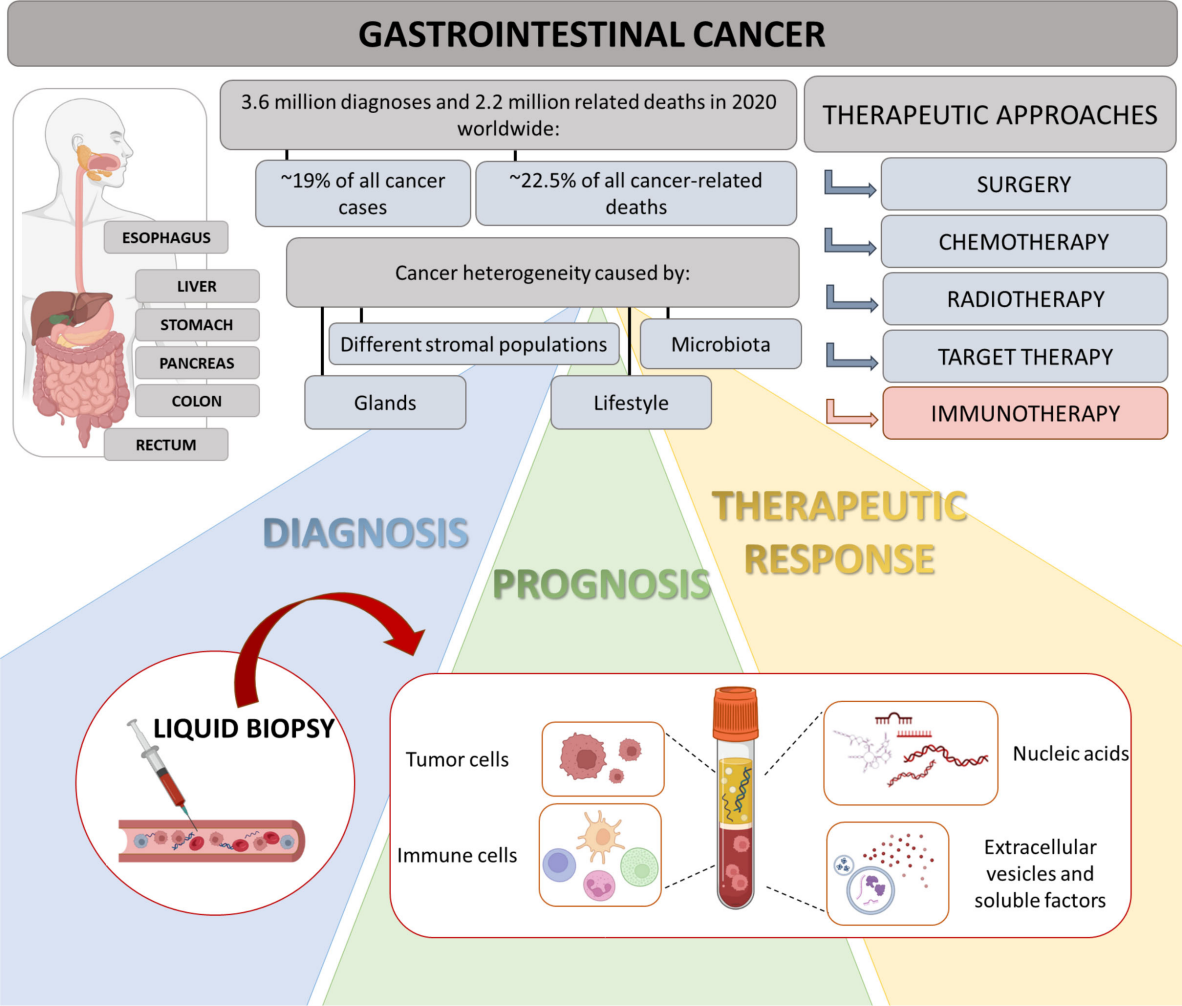
Highlights on GI and role of liquid biopsy. GI cancers represent a group of neoplasms, affecting multiple organs of digestive tract. Due to their heterogeneity, diagnosis is often delayed and progression occurs promptly. Therapeutic approaches include surgery, chemotherapy, targeted therapy and radiotherapy. In last years, immunotherapy has shown an emerging relevance in personalized medicine, considering inter-individual difference in multiple factors, such as genes, environment and lifestyles. In this context, liquid biopsy could represent a valid technology allowing the definition of new putative diagnostic, prognostic and predictive biomarkers for GI patients.

*Tailoring Immunotherapy in Gastrointestinal Cancer: The Role of Circulating Factors* is a Research Topic on the latest advances and future challenges in the field of GI tumors, with a particular focus on circulating factors as biomarker for immunotherapies. Overall, six articles provide interesting new insights regarding the use of immunotherapies in GI tumors, and the effectiveness of potential circulating biomarkers detection for predicting cancer progression or treatment response.

The genetic, molecular and cellular complexity of cancer is now included in fourteen hallmarks of cancer (2). Among them, tumor-promoting inflammation plays a critical role in supporting cancer growth at different levels, by i) providing bioactive molecules to the tumor microenvironment (TME), including growth and survival factors; ii) supporting the development of new blood vessels; iii) activating extracellular matrix-modifying enzymes; iiii) altering the immune response. In particular, the normal hematopoiesis is altered in cancer patients and favors the expansion of pro-tumoral cell components, including tumor-associated macrophages and myeloid-derived suppressor cells, which are able to migrate in the TME and control anti-tumor adaptive and innate immunity (3). Hence, dissecting the heterogeneity of pro-tumoral immune population resident in the tumor tissue, as well as circulating factors, is crucial for better define tumor Immunoscore (4). In this context, the study by Turri et al. retrospectively evaluate the prognostic significance of pre-diagnosis immune profile of resected CRC patients. Interestingly, the authors demonstrate that the immune profile (i.e., leukocytes, lymphocytes, neutrophils, and neutrophil-to-lymphocyte ratio) changes years before CRC diagnosis (at least 24 months before surgery). Moreover, these values are independent prognostic factors for patient overall survival (OS) and cancer-related survival after surgery. As cancer-dependent immune shaping could be easily assessed as routine parameter, the reported data clearly suggest that the longitudinal analysis of pro-inflammatory immune cells may represent a window of opportunity for early cancer detection and surgical patient prognosis.

Despite surgery is one of the main approaches for several GI tumors, not all patients are eligible for surgery, hence require neoadjuvant therapies before surgery or systemic treatments (chemotherapy, radiotherapy, immunotherapy and targeted therapy) (**Figure 1**). Standard treatment of unresectable CRC patients includes different chemotherapeutic drugs (fluorouracil, oxaliplatin, irinotecan) combined with targeted therapy for either vascular endothelial growth factor (VEGF; bevacizumab) or epidermal growth factor receptor (EGFR; cetuximab) (5). Interestingly, the case report discussed by Fan et al. evidences the clinical impact of cetuximab plus chemotherapy (mFOLFOX6) on a patient with rare bone marrow metastasis from rectal cancer, who underwent radical resection. The life expectancy of patients with this stage of the disease, who receive chemotherapy, is no longer than three/six months from the diagnosis: at the time of article submission, the patient treated with mFOLFOX6 chemotherapy plus cetuximab displays an improved long-term stable disease (survival more than eleven months).

Unfortunately, metastatic CRC patients often progress after first- or second-line standard treatment and third-line or post-line treatment is very limited. Fruquintinib (a tyrosine kinase inhibitor, targeting VEGFR1/2/3) and regorafenib (a multi-target kinase inhibitor, targeting VEGFR1/2/3, platelet-derived growth factor receptor and fibroblast growth factor receptor) have been approved for the third-line therapy of metastatic CRC in China. Since clinical trials on the comparison of efficacy and safety between fruquintinib and regorafenib lack, Deng et al. report a retrospective clinical comparison of these two drugs. In particular, the authors demonstrate that the efficacy and toxicity of fruquintinib and regorafenib -administered alone- are similar, and the regimen of regorafenib followed by fruquintinib increase the OS, as compared to the reverse sequence. Moreover, both the drugs synergize with an anti-PD1 inhibitor; nevertheless, in combination with immunotherapy, the median progression-free survival (PFS) and OS of fruquintinib are longer than regorafenib, clearly highlighting the necessity to better define the immunotherapy combinatorial regimens.

In the last ten years, immunotherapy revolutionized the oncology field by the exceptional results obtained in different cancer settings such as melanoma, lung and renal cancers, and DNA mismatch repair-deficient (MMRd)/microsatellite instability-high (MSI) tumors (6). Two case reports contribute to the Research Topic with relevant evidence of immunotherapy applied to GI cancers. The case report provided by Wang et al. highlights the clinical management of immune-related adverse events by the activation of T cell and/or B cell clones against self-antigens. This immune response could explain the bullous pemphigoid (mainly characterized by skin lesions) caused by the anti-PD-1, sintilimab, in a proficient mismatch repair/microsatellite stable CRC patient. Currently, guidelines recommend cessation of immunotherapy for all grades of bullous pemphigoid and administration of drugs to alleviate symptoms. Nevertheless, the case reported here has been treated with oral methylprednisolone (hormone therapy), continuing the combinatorial treatment (sintilimab plus fruquintinib) with the assistance of the dermatologist: patient reached partial response with a PFS of eight months. Saji et al. provide two cases of patients with intrahepatic lymphoepithelioma-like carcinoma (LELCC), an extremely rare subtype of epithelial cholangiocarcinoma. Currently, LELCC is poorly understood, and the main treatment approach remains surgical resection. The two patients affected by LELCC, without Epstein–Barr virus infection, were treated with a combination of the anti-PD-1, nivolumab, with repeated adoptive cell transfer cycles of natural killer–cytokine-induced killer. Interestingly, both patients display a good prognosis with a survival time of more than six years.

Nowadays, tissue biopsy remains the gold standard for tumor identification and diagnosis. However, repeat biopsies to monitor cancer progression are the main obstacles due to their invasive nature and poor patient compliance. Consequently, exploring more feasible approaches could allow to longitudinally analyze disease progression. In this scenario, in the last years the non-invasive liquid biopsy has gained a crucial role in clinical oncology and is now a key part of the modern precise oncology. More than 10 years ago, Pantel and Alix-Panabieres coined the nomenclature “liquid biopsy” to analyze and identify circulating factors in the body fluids (such as blood, urine, saliva) to obtain diagnostic, prognostic, and predictive data (7). Applying cutting-edge technologies, a single blood sample can be processed to simultaneously isolate several analytes, including circulating tumor cells, immune cells, circulating nucleic acids (i.e. circulating tumor DNA, mRNA, long non-coding RNA and microRNA), extracellular vesicles as well as tumor-educated proteins and metabolites, like cytokines (**Figure 1**) (8).

Liquid biopsy assay for clinical management of CRC patients is the main topic of the review by Ceccon et al., in which the authors comprehensively discuss benefits and limitations of the technical aspects and clinical applications of this method. Currently, liquid biopsy in CRC is used for testing *RAS* and *BRAF* as substitute for tumor tissue analysis in stage IV metastatic CRC and profiling *RAS* status in patients resistant to first line anti-EGFR therapies. The authors particularly focus their work on the impact of liquid biopsy in MMRd/MSI CRC patients. Indeed, as MMRd/MSI status has a favorable prognostic value and predicts the response to immunotherapies, it is necessary to establish a non-invasive clinical tool to stratify patients who can actually benefit from the treatment, in case of tissue cannot be collected or discordant information arising from tissue biopsies (i.e., unavailable, insufficient, or inadequate material). Moreover, liquid biopsy-based testing can track resistant subclones during treatment, in order to monitor cancer relapse and progression. Overall, despite liquid biopsy has not reached the clinic yet, evidence clearly supports the potential clinical aspect of this methodology in CRC patients. In particular, liquid biopsy helps to define resected patients who benefit from adjuvant therapy, whereas in metastatic CRC clinicians can monitor immunotherapy response by using liquid biopsy.

Overall, the studies included in this Research Topic highlight the relevance of soluble factors reflecting the immune status of GI cancer patients as diagnostic, prognostic and predictive biomarkers. Consistently, the multiple applications of liquid biopsy could help clinicians to both achieve early diagnosis of cancer and to predict cancer progression and/or treatment response. The use of cutting-edge technologies such as artificial intelligence, single cell analysis and machine learning, in combination with the development of bioinformatic tools, will amplify and standardize the accuracy of liquid biopsy in the near future. Hence, we are strongly convinced that data obtained from the application of circulating factors-based liquid biopsy will profoundly improve the research on innovative therapeutic treatments for GI tumors, in a truly personalized medicine.

## Author contributions

SU: Conceptualization, Funding acquisition, Supervision, Writing – original draft, Writing – review & editing. CB: Conceptualization, Supervision, Writing – original draft, Writing – review & editing. FC: Conceptualization, Funding acquisition, Supervision, Writing – original draft, Writing – review & editing.
